# Blueberry Bioactives as Adjunctive Nutritional Strategies for Pediatric Neurodevelopmental and Emotional–Behavioral Health: Mechanisms, Evidence, and Translational Challenges

**DOI:** 10.3390/nu18132039

**Published:** 2026-06-23

**Authors:** Lina Fan, Shuwei Wei, Xing Yang, Yunmei Ma, Chunting Zhu, Tong Su, Dongfang Shi, Kai Song

**Affiliations:** 1 Special Education College, Changchun University, Changchun 130022, China; 2 Institute of Innovation Science and Technology, Changchun Normal University, Changchun 130032, China; 3 School of Life Science, Changchun Normal University, Changchun 130032, China

**Keywords:** blueberries, anthocyanins, pediatric neurodevelopment, gut–brain axis, neuroinflammation, dietary intervention, emotional–behavioral disorders, functional foods, healthcare translation

## Abstract

The rising prevalence of neurodevelopmental, emotional, and behavioral disorders in children has prompted interest in dietary strategies that target neuroinflammation, oxidative stress, and gut dysbiosis. Blueberries (*Vaccinium* spp.) contain substantial amounts of anthocyanins and other neuroactive polyphenols that may confer neuroprotective effects. We summarize the literature published between 2016 and 2025 to examine how the bioactives in blueberries affect symptoms relevant to children with diagnosed neurodevelopmental or emotional–behavioral disorders, including ADHD, mood problems, and cognitive difficulties. Mechanistically, anthocyanins appear to modulate gut microbial composition, modulate neuroinflammation and alleviate oxidative stress via the Nrf2 pathway, and support synaptic plasticity and neurogenesis. Clinical trials, although limited in number and sample size, have reported modest improvements in mood and verbal memory in typically developing children and adolescents, with some gains in attention and executive function. However, direct trials in children with diagnosed neurodevelopmental or emotional–behavioral conditions remain scarce. There are substantial hurdles to translating these findings. Anthocyanins have poor physicochemical stability and low bioavailability, and routine food processing degrades their activity. Emerging solutions such as green extraction from agricultural by-products, colon-targeted microencapsulation, and zero-waste engineering could address these limitations. Rigorous randomized controlled trials in children with diagnosed neurodevelopmental or emotional–behavioral disorders are essential, as are advances in food engineering. Both are needed to move blueberry-based interventions from the laboratory to application.

## 1. Introduction

Neurodevelopmental, emotional, and behavioral disorders in childhood represent a growing public health and healthcare challenge. In the United States, the prevalence of mental, behavioral, and developmental disorders among children aged 3–17 years increased from 25.3% in 2016 to 27.7% in 2021, with reported increases in anxiety, depression, learning disabilities, developmental delay, and speech or language disorders [1]. Because many mental health difficulties emerge during childhood or adolescence and may persist into adulthood, early supportive strategies are important not only for symptom management, but also for long-term functioning, family well-being, and healthcare resource use [2].

Accessible adjunctive strategies are particularly relevant for children with diagnosed neurodevelopmental disorders (NDDs)—including autism spectrum disorder (ASD), attention-deficit/hyperactivity disorder (ADHD), and intellectual disability—and those with medical complexity [3]. Throughout this review, we use ‘pediatric neurodevelopmental and emotional–behavioral disorders’ to refer to children with clinically diagnosed conditions as defined by DSM-5 or ICD-11 criteria. Children with medical complexity are more likely to experience unmet care needs and fragmented systems of care [3], while unmet mental health needs are also widespread among adolescents [4]. Dietary and lifestyle interventions cannot replace established clinical, psychological, educational, or pharmacological care; however, they may offer scalable and low-risk supportive options when they are evidence-based, acceptable to families, and integrated into broader healthcare and public health strategies [5].

Blueberries (*Vaccinium* spp.) are of interest because they contain anthocyanins, phenolic acids, flavonols, and other polyphenolic compounds with potential relevance to neurobehavioral health [6,7,8]. Beyond direct antioxidant activity, blueberry-derived polyphenols and their metabolites may influence inflammatory signaling, gene expression, microglial activation, and pathways related to neuronal survival and plasticity [9]. Their interaction with the gastrointestinal tract is also important: a substantial fraction of dietary anthocyanins reaches the colon, where microbial metabolism may generate bioactive metabolites and influence gut–brain axis signaling [10]. At the same time, limited stability and low systemic bioavailability remain major translational barriers.

Acute interventions with flavonoid-rich blueberry beverages have improved positive affect in children aged 7–10 years and young adults [11]. Concurrently, double-blind studies have demonstrated that blueberry supplementation can reduce symptoms of depression in a community sample of adolescents. While this evidence is encouraging, it has been generated almost exclusively in typically developing children. Existing reviews on berry polyphenols and brain health have predominantly focused on cognitive aging in older adults [6,12], or the general gut microbiome–brain axis in adult populations [13]. These reviews establish important mechanistic foundations but do not specifically address blueberry interventions for pediatric neurodevelopmental or emotional–behavioral disorders, nor do they evaluate food-engineering barriers to scalable, child-appropriate products. This review addresses three specific questions: (1) What is the strength of current evidence that blueberry bioactives can modulate mood, cognition, and gut microbiota in children, particularly those with neurodevelopmental disorders? (2) What key mechanistic pathways (gut–brain axis, neuroinflammation, synaptic plasticity) plausibly underlie these effects in pediatric populations? (3) What are the principal technological barriers to developing stable, bioavailable blueberry-based functional foods for this population, and how might green engineering and microencapsulation strategies overcome them? This mechanistic plausibility is supported by emerging clinical evidence, albeit primarily from healthy children.

This review synthesizes current evidence on blueberry-derived bioactives in relation to pediatric neurodevelopmental, emotional, and cognitive outcomes. We examine mechanistic pathways, pediatric and translational evidence, formulation and bioavailability challenges, and the implications for healthcare-oriented research. The aim is not to present blueberries as a treatment for neurodevelopmental or psychiatric disorders, but to identify whether blueberry-based dietary strategies merit rigorous evaluation as adjunctive, accessible, and potentially family-centered approaches within preventive and supportive healthcare.

### Literature Search and Selection

This narrative review synthesizes evidence on blueberry bioactives in relation to pediatric neurodevelopmental, emotional, and cognitive outcomes. Literature was identified through searches of the PubMed, Web of Science, and Scopus databases covering the period from January 2016 to March 2025. Search terms included combinations of (“blueberry” OR “Vaccinium” OR “anthocyanin*” OR “berry”) AND (“child*” OR “pediatric” OR “adolescent*” OR “neurodevelopment*” OR “ADHD” OR “autism” OR “depression” OR “anxiety” OR “cognition” OR “memory” OR “mood”) AND (“gut microbiota” OR “microbiome” OR “neuroinflammation” OR “oxidative stress” OR “clinical trial” OR “intervention”). Additional relevant studies were identified through reference lists of key articles and recent reviews.

For the bibliometric analysis presented in Section 2, we used CiteSpace 6.1.R6 software to map co-occurrence networks and temporal trends in the broader field of anthocyanin–gut–brain axis research. The clinical evidence synthesis (Section 5 and Section 6) prioritized human studies in children and adolescents, with mechanistic evidence from adult studies and preclinical models included to establish biological plausibility where pediatric data were lacking. Studies were included if they reported original data on blueberry or anthocyanin interventions, gut microbiota, cognitive or emotional outcomes, or relevant mechanisms. No language restrictions were applied, though the majority of identified studies were in English.

As this is a narrative rather than systematic review, formal risk-of-bias assessment and meta-analysis were not conducted. The aim was to provide a comprehensive, mechanistically grounded synthesis oriented toward healthcare translation and food-engineering applications.

## 2. Research Landscape and Evolution of Hotspots in the Anthocyanin–Gut Microbiota–Brain Health Interface over the Past Decade

**Figure 1:**
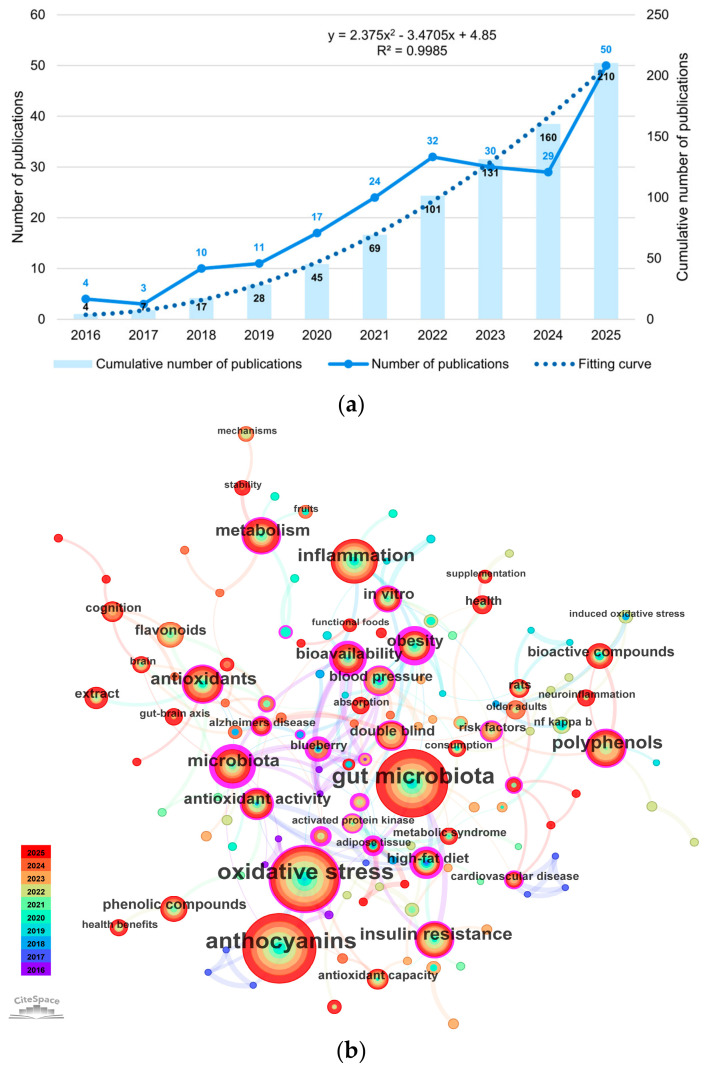

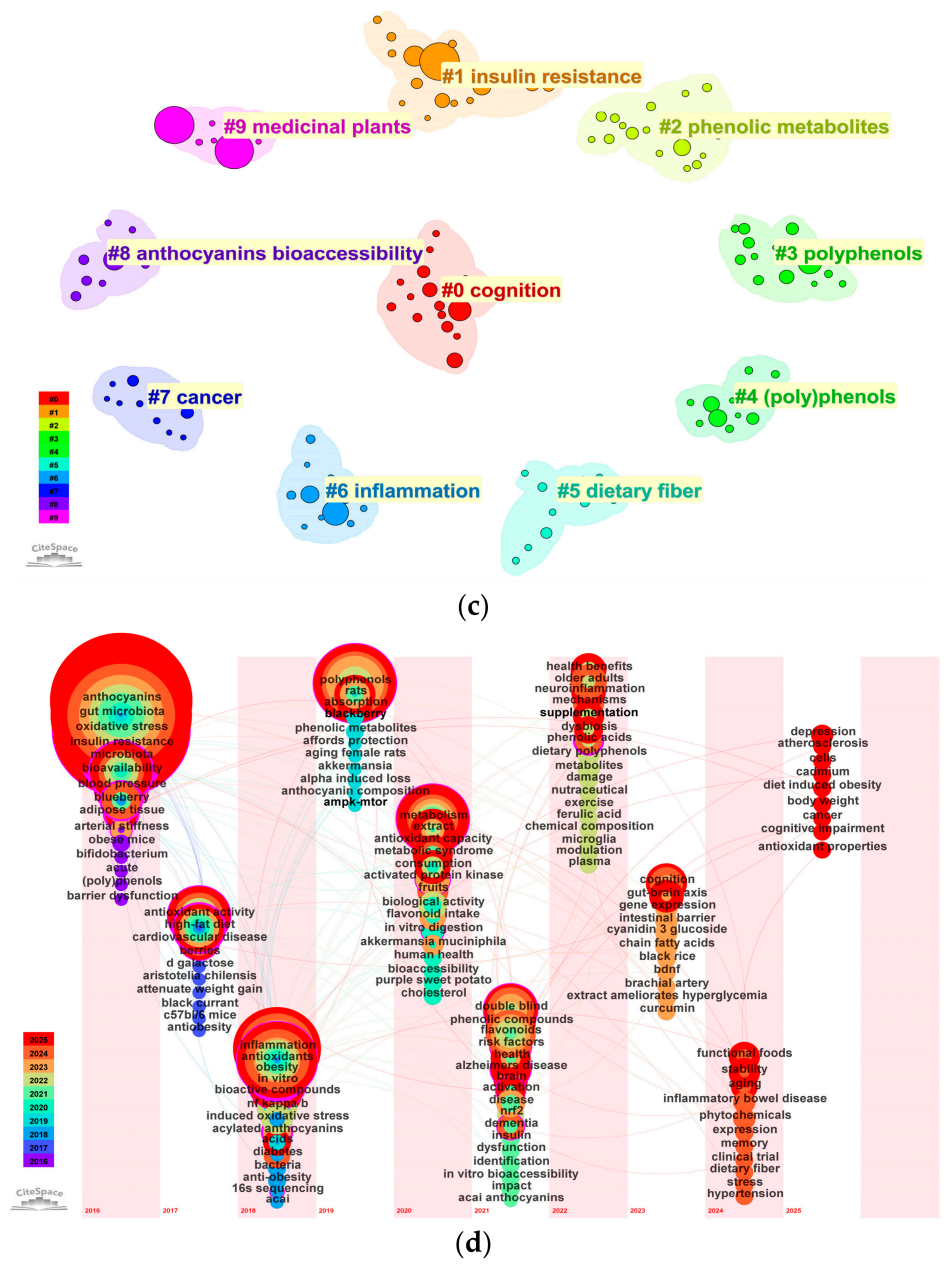
(**a**). Annual publication output and cumulative trend in the anthocyanin–gut microbiota-brain health field from 2016 to 2025. (**b**) Keyword co-occurrence network for anthocyanin−gut−brain axis research. Node size indicates term frequency, and link strength represents co-occurrence intensity. (**c**) Keyword clustering map for anthocyanin−gut−brain axis research. Different colors indicate thematic clusters (Q = 0.6944, S = 0.8619). (**d**) Time-zone evolution map of keywords in anthocyanin−gut−brain axis research, showing the temporal shift from metabolism/oxidative stress to brain health and clinical translation during 2016–2025.

The bibliometric mapping suggests that research on anthocyanins, gut microbiota, and brain health has expanded during the past decade. As shown in Figure 1a, publication output increased between 2016 and 2025; however, data from the most recent year should be interpreted cautiously because database indexing may be incomplete. The keyword co-occurrence network (Figure 1b) shows that “anthocyanins”, “gut microbiota”, “oxidative stress”, “inflammation”, and “bioavailability” are high-frequency nodes, suggesting that current research remains largely centered on the classical pathway linking anthocyanins, gut microecology, inflammation/oxidative stress, and metabolic improvement. At the same time, neuropsychiatric terms such as “cognition”, “neuroinflammation”, “depression”, and “gut–brain axis” also appear in the network. Although their centrality and frequency are not yet prominent, this indicates that the field is shifting from systemic metabolic homeostasis regulation toward brain health interventions, providing a frontier rationale for focusing on ADHD and mood disorders. From the thematic clustering perspective (Figure 1c, network structure Q = 0.6944, S = 0.8619), existing studies can be grouped into four interrelated domains: upstream mechanisms such as gut dysbiosis and oxidative stress; brain health interventions including neuroinflammation suppression, microglial regulation, and improvements in cognition and mood; metabolic transformation and in vitro digestion of anthocyanins; and cross-disciplinary applications such as berry matrices and functional food development. The temporal evolution of keywords (Figure 1d) reflects a clear paradigm shift: between 2016 and 2020, research focused on gut microbiota, peripheral inflammation, high-fat diet, and metabolic syndrome; from 2021 to 2022 onward, nodes such as “Alzheimer’s disease”, “neuroinflammation”, “microglia”, and “gut–brain axis” expanded rapidly; since 2023, frontier terms like “cognition”, “BDNF”, “clinical trial”, and “depression” have emerged, marking the field’s entry into a translational validation phase centered on cognitive function and emotional regulation. In summary, although childhood ADHD and adult mood disorders are not yet the most central topics in the current literature network, the upstream mechanisms that support their pathophysiology, including gut microbiota remodeling, targeted inhibition of neuroinflammation, and counteraction of central oxidative stress, have already accumulated substantial theoretical and empirical foundations.

## 3. Bioactive Components of Blueberries

The potential neurobehavioral relevance of blueberries is not attributable to a single molecule but to a complex class of bioactive components that act synergistically to exert neuroprotective effects [14]. Accumulating evidence indicates that berries can prevent age-related neurodegenerative diseases, improve motor and cognitive function, and also regulate signaling pathways related to inflammation, cell survival, neurotransmission, and enhanced neuroplasticity [9].

### 3.1. A Profile of Neuroprotective Compounds in Blueberries

Blueberries (*Vaccinium* spp.) contain a complex mixture of bioactive compounds rather than a single active molecule. Anthocyanins are among the most studied constituents and include glycosides of cyanidin, delphinidin, malvidin, peonidin, petunidin, and pelargonidin [15]. Other compounds, including phenolic acids and flavonols such as quercetin and myricetin, may also contribute to biological effects through additive or synergistic mechanisms [16]. Accordingly, the neurobehavioral relevance of blueberries should be discussed in terms of a phytochemical matrix and its metabolites rather than as the action of isolated anthocyanins alone. It is important to distinguish between blueberry species and preparation types, as these factors substantially influence anthocyanin content and bioavailability. The most commonly studied species are *Vaccinium corymbosum* L. (highbush or cultivated blueberry, predominant in North America and Asia) and *Vaccinium myrtillus* L. (European bilberry or wild blueberry, predominant in Northern Europe). *V. myrtillus* typically contains 300–700 mg anthocyanins per 100 g fresh weight—approximately 3–5 times higher than *V. corymbosum* (80–150 mg per 100 g)—due to differences in genetics, growing conditions, and fruit size [17,18]. Consequently, studies using *V. myrtillus* may achieve comparable anthocyanin doses with smaller fruit quantities.

### 3.2. Bioavailability, Metabolism, and Central Nervous System Penetration

After ingestion, anthocyanins undergo extensive digestion, absorption, microbial metabolism, and phase-II biotransformation [13]. Some anthocyanin-derived metabolites have been reported in biological compartments relevant to the central nervous system, and preclinical evidence suggests possible accumulation in brain regions involved in cognition, including the hippocampus and cortex [14,19]. However, systemic bioavailability is generally low, and direct central nervous system exposure in children has not been established. Therefore, both direct mechanisms and indirect gut-microbiota-mediated pathways should be considered. In nutritional terms, blueberries provide approximately 57 kcal, 14.5 g carbohydrates, 2.4 g dietary fiber, and 10 g natural sugars per 100 g fresh weight, alongside vitamins C and K, folate, manganese, and potassium. These macronutrients and micronutrients contribute to the overall dietary value of blueberries when consumed as whole fruit, complementing their polyphenol-mediated bioactivity.

## 4. The Neuroprotective Effects of Bioactive Substances in Blueberries

### 4.1. Antioxidant and Anti-Inflammatory Actions

Mechanistic studies provide several biologically plausible pathways through which blueberry-derived polyphenols could influence neurobehavioral outcomes, but most evidence comes from in vitro or animal models. Anthocyanins may activate endogenous antioxidant responses, including the nuclear factor erythroid 2-related factor 2 (Nrf2) pathway, and may affect antioxidant enzymes such as superoxide dismutase and glutathione peroxidase [20,21]. Flavonoids have also been reported to modulate inflammatory signaling, microglial activation, and cytokine production [22]. These pathways are relevant because oxidative stress and neuroinflammation have been implicated in neurodevelopmental and psychiatric conditions. However, the evidence described above is derived primarily from in vitro and animal models, and the extent to which dietary blueberry intake modifies these pathways in children with diagnosed neurodevelopmental or emotional–behavioral disorders remains unknown.

Blueberry polyphenols may also influence neuronal signaling and plasticity. Preclinical studies suggest effects on hippocampal synaptic plasticity, neurogenesis, and intracellular pathways related to learning and memory [9,23]. These findings support mechanistic plausibility, but they should be presented as a rationale for clinical testing rather than as proof of therapeutic benefit in children.

### 4.2. Enhancement of Neuronal Signaling and Plasticity

Beyond cytoprotection, blueberry polyphenols appear to directly modulate synaptic function and neuronal connectivity. Preclinical studies demonstrate that supplementation enhances hippocampal synaptic plasticity, the activity-dependent modification of synaptic strength underlying learning and memory consolidation [23]. Animal models demonstrate that blueberry consumption can increase neurogenesis, particularly in the hippocampus, and modulate critical neuronal signaling pathways involved in cell survival and growth. These results suggest that blueberry bioactives can foster a brain environment that is more resilient and more efficient at processing information [9].

### 4.3. Neuroprotection Mediated by Gut Microbiota

**Figure 2:**
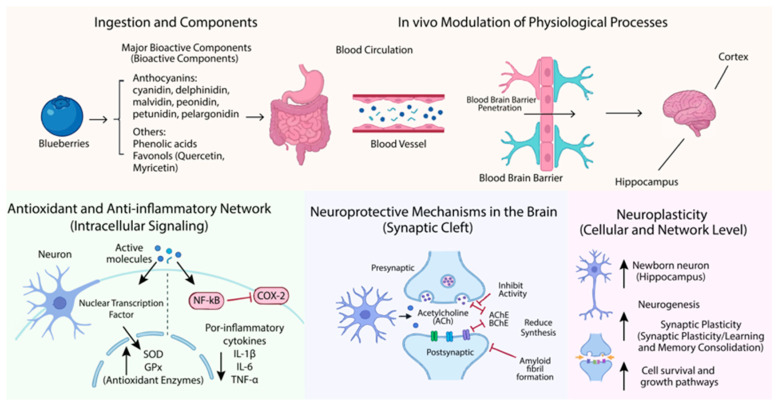
The in vivo processes and neuroprotective mechanisms of active ingredients and their metabolites in blueberries.

Anthocyanins, a class of water-soluble flavonoids that are widely present in berries such as blueberries, have been implicated in the regulation of central nervous system function through the gut microbiota–gut–brain axis, including the modulation of tryptophan metabolism and short-chain fatty acid production [13]. Animal experiments provide further evidence. For example, Li et al. administered 300 mg/kg blueberry mulberry extract via gavage to 18 month old mice daily for 6 weeks [24]. The results showed improved cognitive performance and reduced neuronal loss, decreased levels of pro-inflammatory cytokines (IL-6, TNF-α) in the brain and intestines, and elevated expression of intestinal tight junction proteins (ZO-1 and occludin). These findings indicate that berry-derived bioactive compounds may alleviate cognitive impairment and suppress neuroinflammation by reshaping gut microbiota and their metabolites (Figure 2).

## 5. Pediatric Evidence for Blueberry Bioactives: Gut Microbiota, Mood, and Cognition

### 5.1. Intervention and Impact on Gut Microbiota

The journey of blueberry bioactives to the colon initiates a cascade of interactions that reshape the gut ecosystem. Consuming blueberries as an early complementary food may alleviate allergic symptoms, modulate immune biomarkers, and promote beneficial shifts in the gut microbiota during infancy, but the specific bioactive components in blueberries responsible for these effects remain unidentified [17]. Current evidence suggests that anthocyanins and blueberry extracts can have significant proliferative effects on promoting health, most notably in *Bifidobacterium* spp. and *Lactobacillus* spp. [25]. For instance, the results of one 4-week intervention suggested that blueberry provided the most benefit during the more difficult aspects of the task. No significant blueberry-related effects were observed on the auditory verbal learning task or the child’s version of the positive and negative affect schedule. Urinary metabolite analyses indicated significant increases in different metabolites in blueberry and placebo groups after 4 weeks of consumption [26]. In addition to promoting beneficial bacteria, anthocyanins also appear to exert an inhibitory effect on certain potentially pathogenic or pro-inflammatory microbes. In vitro studies have shown that anthocyanins can inhibit the growth of species like *Clostridium histolyticum*, a known pathogen [25]. More broadly, berry extracts have demonstrated antimicrobial and anti-adhesion properties against a range of pathogenic bacteria, suggesting that they can help to create a less inflammatory gut environment by selectively suppressing the growth of undesirable organisms [27]. A compelling example is provided by Horasan Sagbasan et al., who conducted a randomized controlled trial in healthy children aged 7–10 years and found that after four weeks of supplementation with freeze-dried wild blueberry powder, the relative abundance of Bacteroidetes in the children’s gut microbiota significantly increased, while that of Firmicutes significantly decreased, leading to an elevated Bacteroidetes/Firmicutes ratio—a ratio often considered an indicator of gut health [28]. Children in the intervention group showed significant improvements in executive function compared to the placebo group. Low Bacteroidetes/Firmicutes ratios have frequently been observed in children with autism spectrum disorder, and have been correlated with behavioral abnormalities. This finding suggests that blueberries may indirectly influence cognitive function through modulation of the gut microbiota. By promoting beneficial genera while inducing favorable phylum-level shifts, these targeted effects provide a plausible link between the dietary intervention and potential health benefits [29]. In an animal model study of autism spectrum disorder, Serra et al. used a valproic acid-induced mouse model to simulate autism-like phenotypes and found that intervention with an anthocyanin-rich extract derived from Portuguese blueberries was associated with reductions in autism-like behaviors. The findings also suggested that these effects were accompanied by decreased intestinal inflammation and neuroinflammation, and normalization of gut microbiota structure [30].

### 5.2. The Impact on Emotional Disorders

The elevated risk of depression among children with neurodevelopmental disorders is well-substantiated by epidemiological evidence, with prevalence rates significantly exceeding those observed in the general child population. This group of people has a significantly increased susceptibility to emotional disorders [31]. Consumption of wild blueberries has become a promising way to support the mental health of children and adolescents; however, adolescents may be more sensitive to emotional changes than children, partly because adolescence is a particularly sensitive period of development, and the growth of the prefrontal cortex can alter emotions and executive function [32]. Clinical trial evidence has demonstrated that a single dose of flavonoid-rich blueberry can elicit significant increases in positive affect in children aged 7–10 years within two hours post-ingestion, suggesting a rapid modulation of mood states. Although such acute effects are typically transient, chronic supplementation strategies have shown greater promise for addressing persistent symptomatology [11]. A 4-week parallel-group randomized controlled trial involving 50 adolescents (aged 12–17 years; with subclinical depressive symptoms, BDI-II score ≥ 14) found that daily supplementation with wild blueberry powder (*Vaccinium angustifolium*, 250 g fresh weight equivalent, approximately 287 mg anthocyanins) significantly reduced depressive symptoms compared to a placebo, as measured by the Beck Depression Inventory. These therapeutic benefits are hypothesized to operate through multiple mechanisms, including enhanced cerebral blood flow to regions implicated in emotional regulation and the inhibition of monoamine oxidase, thereby preserving neurotransmitter availability. Nevertheless, preliminary data from children and adolescents indicate that while chronic intake reliably improves executive functions such as attention and memory, detectable mood changes may necessitate extended intervention durations or larger sample sizes to achieve statistical and clinical significance.

Evidence for the acute enhancement of positive mood by blueberry flavonoids is consistent and robust across both children and young adult populations. Multiple studies have reported that consuming or supplementing blueberries at varying doses and durations can improve positive emotions [12,33]. Critically, direct long-term trials in clinically diagnosed pediatric depression appear to be lacking. All existing studies have been restricted to healthy or subclinical cohorts, thereby limiting the direct applicability of these findings to the population of interest.

### 5.3. The Impact on Memory and Cognition

A substantial body of research has examined the cognitive effects of blueberries in healthy adults, and emerging studies in typically developing children show promise. However, direct evidence in children with diagnosed neurodevelopmental or emotional–behavioral disorders remains virtually absent. Existing clinical trials have predominantly focused on healthy, school-aged children, typically between the ages of 7 and 10 [34], which is a critical period for frontal lobe maturation and executive function development. These studies provide preliminary evidence for the effects of blueberry supplementation on memory and cognition in typically developing children [12,35], though whether such benefits extend to children with neurodevelopmental conditions remains unknown and requires dedicated investigation.

#### 5.3.1. Effects on Memory

Blueberries have tangible benefits during periods of cognitive development in young people and cognitive decline in the elderly [36]. Supplementation with wild blueberry has demonstrated benefits for verbal memory, as assessed by tasks such as the Auditory Verbal Learning Test [34,35,36,37]. In acute intervention studies, children who consumed a single dose of blueberry beverage showed modest but statistically significant improvements in immediate verbal memory performance shortly after consumption [38]. Effects were also noted for delayed recall, a measure of memory consolidation, with blueberry groups outperforming their placebo counterparts. Some studies have reported enhanced performance in total memory acquisition across multiple learning trials and in short-delay recall [34,39]; these findings have been interpreted as suggesting more efficient encoding and retention of verbal information.

#### 5.3.2. Attention and Processing Speed

Previous studies in typically developing children have reported that blueberry supplementation leads to faster reaction times on attention tasks [40]. Using the Modified Attention Network Task, significant improvements in executive function, specifically faster reaction times, were observed following acute supplementation with wild blueberry relative to the placebo [41,42]. These gains in processing speed occurred without a corresponding decrease in accuracy, suggesting enhanced cognitive efficiency rather than a speed–accuracy trade-off. These results suggest that blueberry bioactives may support the efficiency with which children process and respond to visual information.

#### 5.3.3. Dose–Response and Time-Course Effects

The available evidence suggests a dose–response relationship for the cognitive effects of blueberries in children. Studies comparing different doses have found that a higher anthocyanin dose consistently yields superior cognitive performance compared to a lower dose or a placebo. The effects observed are acute, meaning that they manifest within hours of a single dose. The timing of these cognitive improvements aligns with the known pharmacokinetics of polyphenol metabolism, with benefits appearing at time points corresponding to peak concentrations of metabolites in the bloodstream, typically between 1.15 and 6 h after consumption. This temporal link indicates a plausible physiological link [34].

## 6. The Potential Mechanism of Bioactive Components in Blueberries in Children’s Neurological and Mental Health

### 6.1. Characteristics of Gut Microbiota in Neurodevelopmental Disorders and Potential Intervention of Blueberries

Growing evidence suggests that imbalance in the composition and function of the gut microbiota is an important factor in the pathophysiology of a range of neurological, psychiatric, and neurodevelopmental disorders [43]. Neurodevelopmental disorders (NDDs) represent a class of complex conditions that manifest early in childhood and are characterized by impairments in personal, social, academic, or occupational functioning. Among the most prevalent of these are autism spectrum disorder (ASD) and ADHD. In children with ASD, the reported prevalence of gastrointestinal issues, including chronic constipation, diarrhea, abdominal pain, and flatulence is substantially higher than that observed in neurotypical individuals [17]. Critically, the connection between gut health and neurological function in these children is not merely correlational. Multiple studies have observed a direct association between the severity of GI symptoms and the intensity of core ASD manifestations. This consistent clinical observation suggests that the gut is not simply a site of peripheral comorbidity, but may be mechanistically intertwined with the central pathophysiology of the disorder. This transforms the gastrointestinal tract from a secondary concern into a central therapeutic target, where interventions aimed at restoring gut homeostasis could plausibly yield downstream benefits for behavior and cognition [44]. In children with ASD accompanied by symptoms of constipation, studies have also confirmed alterations in gut microbiota composition, including decreased concentrations of acetate and butyrate in the stool, alongside elevated valerate levels [45]. In a study involving 18 children, two-year follow-up results demonstrated that improvements in gastrointestinal symptoms were sustained, with further improvements observed in autism-related symptoms [46].

**Figure 3:**
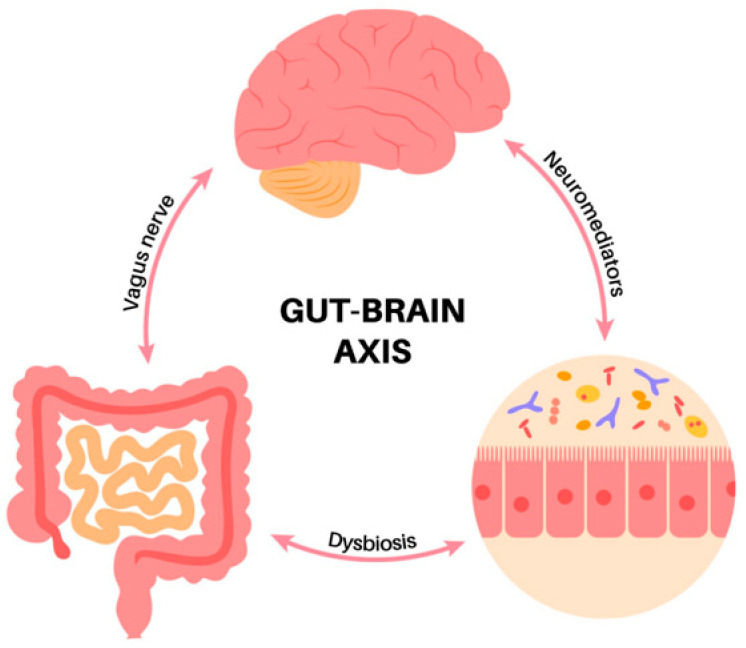
Blueberry extract modulates gut microbiota to improve symptoms of neurodevelopmental disorders.

The establishment of the gut microbiota occurs in early life, a period that is highly dynamic and malleable, coinciding with critical windows of brain development and immune system maturation (Figure 3) [29]. This temporal overlap suggests that early-life environmental factors, particularly diet, can have a profound and lasting impact on the trajectory of neurodevelopment. Factors known to influence the infant microbiome, such as mode of delivery and feeding method, have been associated with NDD risk. For instance, breastfeeding for more than six months is associated with a lower risk of developing ASD and may protect against GI symptoms in high-risk infants, pointing to a protective role for the specific microbial signatures established by human milk [47]. These findings highlight infancy and early childhood, particularly the complementary feeding period when freeze-dried blueberry powder is introduced, as periods with the potential for having long-term effects on gut microbiota development [48].

### 6.2. Antioxidant and Anti-Inflammatory Neuroprotective Mechanisms Based on Model Conditions

The brain exhibits a unique susceptibility to oxidative stress, defined as an imbalance between the production of damaging reactive oxygen species (ROS) and endogenous antioxidant capacity [49]. Chronic oxidative stress and its associated neuroinflammatory sequelae are now recognized as central pathological drivers in age-related cognitive decline and neurodegenerative disease [50]. Blueberry polyphenols and their metabolites exert potent protective effects by directly addressing these processes. They function as powerful antioxidants, capable of directly scavenging ROS and chelating metal ions that catalyze their formation. Studies in animal models demonstrate that supplementation can reduce the activation of microglia and decrease the expression of pro-inflammatory cytokines such as tumor necrosis factor-alpha (TNF-α) and interleukin-1 beta (IL-1β). This dual action of mitigating oxidative damage and quelling neuroinflammation creates a more favorable cellular environment for optimal neuronal function and survival [51]. In vivo experiments in aged mice have shown that high-dose anthocyanin treatment increases brain dopamine levels [52] and alleviates anxiety-like behaviors in the elevated plus-maze test [53,54]. Antidepressant-like effects have also been observed, comparable to those observed with imipramine [55] in specific tasks. However, extrapolation of these findings to humans is limited by differences in dopaminergic systems between species and the acute, high-dose protocols used in animal studies. Chronic treatment with wild blueberry extract has been shown to improve episodic memory and reduce systolic blood pressure in older adults [56]. These findings require confirmation in randomized controlled trials before conclusions can be drawn about anthocyanins’ effects on human mood disorders.

### 6.3. The Regulatory Mechanism of Synaptic Plasticity and Neurogenesis

Beyond the antioxidant and anti-inflammatory effect documented in preclinical models, blueberry bioactives may enhance neurobiological mechanisms underlying learning and memory. In vitro and animal studies indicate that blueberry polyphenols can modulate intracellular signaling cascades implicated in synaptic plasticity. These effects include increased expression of neurotrophic factors, particularly brain-derived neurotrophic factor and nerve growth factor [57,58]. Brain-derived neurotrophic factor is essential for long-term potentiation, a cellular correlate of synaptic strengthening, and plays roles in neuronal growth and differentiation in animal models [59]. Whether these mechanisms operate similarly in children following dietary blueberry consumption remains to be determined. Active ingredients in blueberries may promote neurogenesis, the generation of new neurons, a process largely restricted to the dentate gyrus of the hippocampus in adulthood. In vitro studies using cultured adult human hippocampal neural progenitor cells demonstrate that blueberry extract treatment enhances cell viability and proliferation rates [60]. Animal studies in aged mice have shown increased hippocampal neurogenesis following supplementation [61]. These findings derive from in vitro and preclinical models and suggest potential effects on synaptic plasticity and hippocampal function. However, whether these mechanisms operate similarly in children with neurodevelopmental or emotional–behavioral disorders following dietary blueberry consumption remains to be determined through rigorous clinical investigation. Mechanistic studies also suggest that blueberry bioactives may influence neurodevelopment through multiple pathways, including the mitigation of oxidative stress and neuroinflammation, as well as the modulation of synaptic plasticity and neurogenesis. However, further research is needed on the clinical application of blueberry supplements, especially in terms of formulation, dosage, and translational feasibility, as well as the in-depth development of alternative products. These issues pose both challenges and opportunities for future research.

## 7. Current Applications and Challenges

### 7.1. Comparative Bioactive Profiles of Berry-Derived Polyphenols

Although this review focuses on blueberries, we will briefly compare grapes, strawberries, and pomegranates in this section for three reasons: (1) to validate shared neuroprotective pathways (gut microbiota modulation, anti-inflammatory signaling) across berry types [62,63,64,65,66,67]; (2) to highlight unique bioactives (resveratrol, ellagitannins, urolithins) that may inform multi-fruit formulations [68,69,70,71,72,73,74]; and (3) to contextualize blueberries within the broader landscape of berry-based interventions for pediatric neurodevelopmental health. To develop more comprehensive therapies, it is essential to identify and explore alternative functional fruits, such as grapes, strawberries, and pomegranates, and assess their effects in specific neurodevelopmental and emotional–behavioral disorders. A comparative analysis of these alternative fruits can reveal whether they share common neuroprotective pathways with blueberries, while also offering unique biological or practical advantages. Ultimately, understanding these shared mechanisms and distinct benefits will provide crucial scientific insights for designing broader, more effective, and adaptable dietary intervention strategies for children.

**Figure 4:**
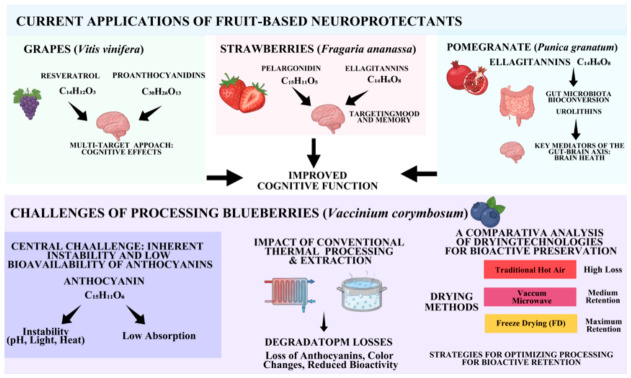
Current applications of fruit-derived bioactives in gut–brain axis modulation and the physical–chemical challenges in processing anthocyanin-rich blueberries.

Grapes contain flavonoids, phenolic acids, anthocyanins, and resveratrol, which exhibit antioxidant, anti-inflammatory, and neurotrophic properties in preclinical models; they may also cross the blood–brain barrier, though this remains unconfirmed in humans [62,63,64,65]. Many grape polyphenols have poor absorption in the upper intestine and may exert local effects upon reaching the colon, where they can regulate gut microbiota composition and increase short-chain fatty acid production as signaling molecules in gut–brain communication [66,67]. Strawberries are rich in pelargonidin and ellagitannins; in overweight adults with insulin resistance, 12 weeks of freeze-dried strawberry powder reduced memory interference and depressive symptoms, but its effects in children are unknown [68,69]. Pomegranate presents a unique metabolic pathway in which colonic microbiota convert ellagitannins to urolithins, compounds that inhibit neuroinflammation, prevent β-amyloid fibrillation, and protect against neuronal apoptosis in preclinical studies. Pomegranate also acts as a prebiotic, increasing beneficial bacteria such as *Bifidobacterium*, *Lactobacillus*, *Roseburia*, and *Faecalibacterium* [70,71,72,73,74]. Despite these mechanistic parallels, blueberries remain distinguished by their high anthocyanin content and comparatively robust clinical evidence base in cognitive aging [13,24]. The following section examines technical challenges specific to blueberry processing, with implications generalizable to broader berry-based formulations for children with neurodevelopmental or emotional–behavioral disorders (Figure 4).

### 7.2. Building a Sustainable Food-Engineering System for Mental Health

Translating the unique neuroprotective potential of anthocyanins from laboratory mechanisms into daily functional carriers that are widely accessible and economically affordable is a central challenge for modern food engineering [18,75]. To achieve socially inclusive and ecologically sustainable public mental health intervention, food scientists and engineers must systematically address multiple limitations affecting anthocyanins during physicochemical processing and in vivo metabolism [76], including dose standardization, child acceptability, safety, low-glycemic formulation, accessibility and cost, and clinical trial reproducibility. These healthcare-anchored targets determine whether anthocyanin-based interventions can progress to scalable, evidence-based nutritional therapeutics for children with neurodevelopmental or emotional–behavioral disorders.

#### 7.2.1. Dual Barriers of Processing Instability and Low Bioavailability

Anthocyanins exhibit pronounced physicochemical fragility during conventional food processing, storage, and distribution [18,77]. Thermal load, photooxidative stress, and sharp fluctuations in matrix pH can induce irreversible ring opening and degradation of their core carbon skeleton, leading to loss of antioxidant and central anti-inflammatory activity [18,77,78]. As shown in Table 1, different drying technologies involve a clear trade-off between active compound retention and environmental energy consumption. Hot air drying (HAD) is inexpensive but causes the most severe thermal damage to anthocyanins. Freeze drying (FD) can nearly maximize the preservation of bioactives, but its high operating energy demand and equipment cost conflict with low-carbon manufacturing. In contrast, ultrasound-assisted vacuum drying (US-VD) enhances mass transfer through acoustic cavitation and provides a more sustainable compromise between activity retention and reduced energy consumption.

**Figure 5:**
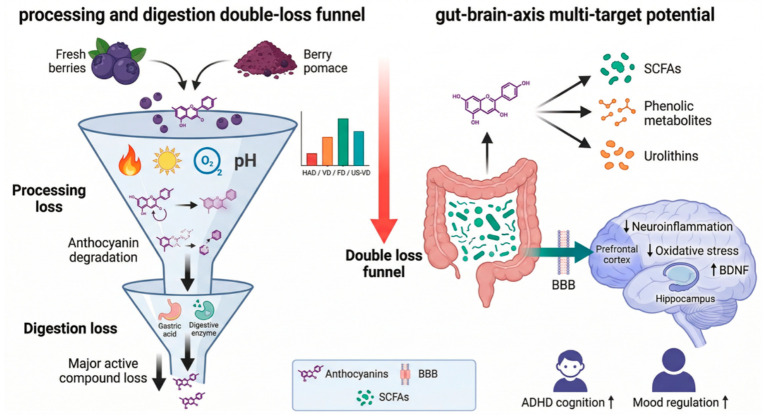
Schematic representation of the processing–digestion double-loss challenge and multi-target neuroprotective potential of berry bioactives in gut–brain axis intervention.

Even after processing barriers are addressed, anthocyanins still face stringent bioavailability constraints in vivo [18,79]. Unprotected free anthocyanins undergo extensive degradation in the highly acidic gastric environment and under upper-gastrointestinal enzymatic exposure, resulting in substantial loss before they reach the key target site, the colon [75,79]. Figure 5 illustrates the processing–digestion double-loss funnel faced by anthocyanins and other bioactives when crossing the gut–brain axis intervention route. This physicochemical bottleneck provides a direct technological rationale for advanced delivery systems [75].

**Table 1:** Comparison of the effects of different food drying technologies on anthocyanin stability and sustainability aspects.

Drying Technology	Core Process Principle	Anthocyanin Retention	Total Phenol Retention	Key Processing and Sustainability Advantages	Main Environmental/Economic Limitations	References
Hot air drying (HAD)	Moisture evaporation through convective heat transfer	Lowest	Marked loss	Very low equipment investment; simple operation	Long drying duration causes severe thermal damage and pigment degradation	[78]
Vacuum drying (VD)	Moisture evaporation under reduced pressure	Moderate	Moderate retention	Low temperature reduces oxidation risk; moderate operating cost	Higher cost than HAD; possible structural collapse	[80]
Freeze drying (FD)	Ice sublimation under deep vacuum	Highest	Maximum retention	Excellent preservation of bioactives, color, and porous microstructure	High energy demand and equipment investment; limited sustainable scalability	[18,80]
Ultrasound-assisted vacuum drying (US-VD)	Acoustic cavitation generates microchannels to assist vacuum evaporation	Relatively high	High retention	Shortens drying time and carbon footprint; reduces thermal damage	Complex hybrid equipment; higher initial capital investment than conventional processes	[18,78]

#### 7.2.2. Green High-Value Utilization of Agricultural By-Products: Implementing a Zero-Waste Circular Economy

In conventional linear fruit and vegetable processing, such as juice or jam production, large amounts of anthocyanin-rich berry pomace and peels are commonly categorized as low-value waste and disposed of through landfill [81,82]. This crude disposal method not only wastes valuable natural polyphenol resources, but also contributes to greenhouse gas emissions and solid waste burdens in soil during decomposition, contradicting circular-economy principles [74,81,82].

To address this contradiction, modern green food engineering aims to establish high-value recovery chains for agricultural by-products. For example, novel green separation systems with low dependence on organic solvents, such as aqueous two-phase extraction (ATPE), can recover high-purity anthocyanins from blueberry residues with a reduced carbon burden [82]. Studies further show that directionally fermented berry pomace not only retains a strong antioxidant matrix, but can also remodel host gut microbial communities more effectively than crude raw materials and amplify the production of neuroprotective SCFAs [81]. High-value “resetting” of processing by-products can therefore lower the basal raw material cost of functional foods while improving the ecological resilience and sustainability of the entire food supply chain [74,82].

#### 7.2.3. Overcoming Delivery Barriers: Advanced Colon-Targeted Microencapsulation Systems

Advanced targeted delivery technologies, especially microencapsulation, are one of the most promising methods of protecting anthocyanin molecular structures from both thermal processing stress and harsh gastrointestinal environments [75,79]. As summarized in Table 2, natural polysaccharides, plant proteins, and biodegradable lipids can serve as food-grade wall materials to precisely embed anthocyanins in micro- or nano-scale compartments, thereby providing strong thermodynamic and chemical barrier protection for the core material [75,78].

More importantly, these intelligent delivery systems enable the spatiotemporal programming of release kinetics [79]. They can resist acid- and enzyme-mediated degradation in the upper gastrointestinal tract and ensure that the active payload reaches the distal colon largely intact [18,75,79]. At the target site, wall materials are degraded by enzymes produced by commensal gut bacteria, releasing concentrated anthocyanins for microbial fermentation and thereby fully activating the neuroregulatory cascade described above [18,79]. With these delivery strategies, engineers can reduce dependence on cold-chain logistics and develop shelf-stable, child-appropriate formulations such as sugar-free gummies, thereby overcoming the final translational barrier from laboratory research to household use [18,75].

#### 7.2.4. Constructing a Zero-Waste Sustainable Food-Engineering Closed-Loop Model

**Figure 6:**
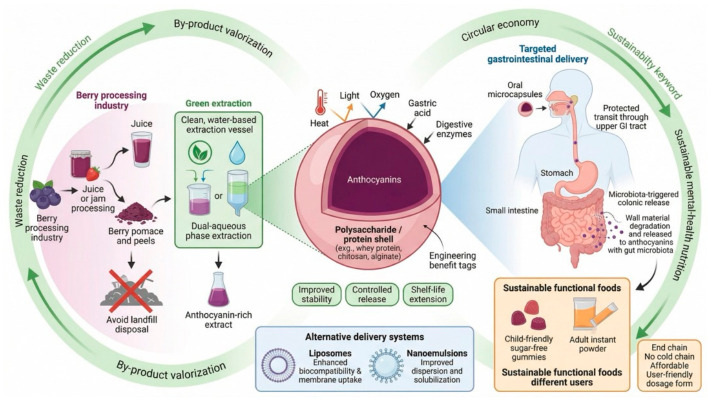
Zero-waste closed-loop berry processing and targeted microencapsulation engineering scheme for public mental health.

Figure 6 presents a closed-loop model that integrates high-value agricultural solid-waste utilization with targeted microencapsulation technology. This model moves away from conventional linear consumption. At the upstream end, green water-based extraction captures bioactive fractions from processing residues while simultaneously addressing resource depletion and environmental burden. At the midstream stage, natural macromolecules are used for intelligent encapsulation. At the downstream stage, the system generates new health products that are independent of cold-chain logistics and capable of colon-targeted delivery. This closed-loop paradigm enables the scalable supply of high-value bioactives with a low environmental footprint and reduces the final market premium of the product, allowing individuals with special needs and low-income families to afford long-term use. It therefore provides a practical technical template for transforming the food industry toward combined environmental welfare and public health benefits.

### 7.3. Safety, Tolerability, and Considerations for Clinical Use in Children

Safety considerations are paramount when evaluating dietary interventions for children, particularly those with neurodevelopmental or emotional–behavioral disorders who may be receiving pharmacological treatment. Blueberries consumed as whole fruit or minimally processed foods are generally recognized as safe and well-tolerated in pediatric populations, with a long history of dietary use and low allergic potential [17,48]. Clinical trials in healthy children have not reported serious adverse events associated with blueberry consumption at doses ranging from approximately 200 to 500 mg anthocyanins per day [34,35,41].

However, several considerations warrant attention when translating findings to children with diagnosed neurodevelopmental or emotional–behavioral disorders. First, concentrated anthocyanin extracts or encapsulated formulations differ from whole-food consumption and may deliver higher polyphenol doses than would be obtained through normal dietary intake. Although anthocyanins exhibit low acute toxicity in preclinical models, pediatric dose–response data for concentrated supplements remain limited, and age-appropriate intake levels have not been formally established [18,75]. Second, potential drug-nutrient interactions should be considered, particularly in children receiving pharmacotherapy. For example, children with ADHD commonly receive stimulant medications such as methylphenidate or amphetamine derivatives. While no direct interactions between blueberry anthocyanins and these agents have been reported, polyphenols can theoretically modulate cytochrome P450 enzymes and affect drug metabolism [62]. Similarly, children receiving selective serotonin reuptake inhibitors (SSRIs) for anxiety or depression may theoretically be at risk for additive effects if anthocyanins modulate monoamine oxidase activity, as suggested by some preclinical studies [52,55]. These interactions remain speculative and require systematic pharmacokinetic investigation, but they underscore the importance of informing healthcare providers when introducing concentrated polyphenol supplements. Third, gastrointestinal tolerance and sugar content are practical concerns. Some children with autism spectrum disorder or other neurodevelopmental conditions experience chronic gastrointestinal symptoms [44], and the introduction of new dietary components should be gradual and monitored. Whole blueberries and some commercial blueberry products contain natural sugars; formulations intended for long-term use in children should ideally be low-glycemic and sugar-free to avoid contributing to excessive energy intake or dental caries [18].

Finally, it is critical to distinguish between blueberries as part of a healthy, varied diet and the use of concentrated supplements as adjunctive therapeutic interventions. Whole-food consumption within age-appropriate serving sizes is unlikely to pose safety concerns and aligns with the general dietary recommendations for children. However, the use of standardized, concentrated anthocyanin supplements in children with diagnosed disorders should be considered investigational and undertaken only within the context of clinical trials or under medical supervision, with careful monitoring for tolerance, adherence, and potential interactions with concurrent treatments.

In summary, while blueberry-derived bioactives appear safe in the context of normal dietary consumption, their use as concentrated adjunctive interventions in children with neurodevelopmental or emotional–behavioral disorders requires careful consideration of dose, formulation, concurrent medications, individual tolerance, and integration within comprehensive care plans.

## 8. Conclusions

Current evidence suggests that blueberry-derived anthocyanins and related polyphenols have biologically plausible mechanisms relevant to pediatric neurobehavioral health, including the modulation of gut microbiota, oxidative and inflammatory signaling, and pathways involved in synaptic plasticity. Pediatric clinical studies in typically developing children and community adolescent samples have reported improvements in selected mood and cognitive outcomes. However, direct evidence in children with clinically diagnosed neurodevelopmental or emotional–behavioral disorders remains scarce. To advance this field from mechanistic plausibility to clinical evidence, future research should prioritize rigorously designed randomized controlled trials in children with diagnosed neurodevelopmental or emotional–behavioral disorders. We propose the following operationalized parameters:

Target population: Children aged 6–12 years with clinically confirmed diagnoses (e.g., ADHD according to DSM-5 criteria, ASD with documented social communication deficits, or mood disorders with validated screening scores), recruited from pediatric neurology, psychiatry, or developmental pediatrics clinics. Inclusion criteria should document concurrent pharmacological and behavioral treatments to enable the evaluation of adjunctive effects and potential interactions.

Intervention and dose: Standardized blueberry preparations with verified anthocyanin content, preferably freeze-dried whole-fruit powder or validated extracts. Recommended dose range: 200–500 mg total anthocyanins per day (approximately equivalent to 1–2 cups fresh blueberries or 15–30 g freeze-dried powder), delivered in child-appropriate, low-sugar formulations. Studies should clearly report blueberry species (*Vaccinium corymbosum* or *V. myrtillus*), harvest conditions, processing methods, and lot-to-lot variability in bioactive content [18,75].

Duration and follow-up: Minimum intervention duration of 8–12 weeks to allow for gut microbiota remodeling and stabilization of cognitive or mood effects, with assessment time points at baseline, mid-intervention, end of intervention, and at least 4 weeks post-intervention to evaluate persistence of benefits.

### Outcome Measures

For ADHD: parent- and teacher-rated Conners 3 scales, continuous performance tests (e.g., CPT-3), and executive function batteries (e.g., BRIEF). For mood and emotional regulation: Child Depression Inventory (CDI-2), Positive and Negative Affect Schedule for Children (PANAS-C), and anxiety scales (e.g., SCARED). For cognition: Auditory Verbal Learning Test (AVLT) for verbal memory, Modified Attention Network Task (MANT) for attention and executive function, and processing speed measures. For mechanistic endpoints: gut microbiota profiling (16S rRNA sequencing), fecal short-chain fatty acids (acetate, propionate, butyrate), peripheral inflammatory markers (IL-6, TNF-α, CRP), and if feasible, blood anthocyanin metabolites to verify compliance and bioavailability.

Study design features: Double-blind, placebo-controlled, parallel-group design with stratification by diagnosis, age, and medication status. Sample size should be determined by power calculation based on clinically meaningful effect sizes (e.g., Cohen’s d ≥ 0.4 for primary outcomes). Intention-to-treat analysis, detailed reporting of adherence (e.g., returned sachets, dietary logs), adverse events, and concomitant treatments are essential. Studies should also include acceptability and feasibility measures to inform real-world implementation [5].

In summary, while current evidence establishes mechanistic plausibility and preliminary cognitive benefits in typically developing children, blueberry-based dietary strategies should be regarded as investigational adjunctive approaches for children with neurodevelopmental or emotional–behavioral disorders. Rigorous clinical trials using the parameters outlined above, coupled with advances in food engineering to address bioavailability and stability challenges, are essential to determine whether these strategies can meaningfully contribute to comprehensive, family-centered care.

## Figures and Tables

**Table 2:** Comparison of the characteristics of advanced food-grade delivery systems designed to overcome the anthocyanin biobarrier.

Delivery Technology	Common Food-Grade Wall Materials	Core Food-Engineering and Physical Advantages	Translational Potential in Gut–Brain Axis Intervention	References
Microencapsulation	Proteins such as whey protein; polysaccharides such as chitosan and alginate	Builds a robust thermodynamic barrier; markedly improves baking and light stability; broadly applicable processing cost	Resists gastric acid and enables colon-targeted release for microbial fermentation and SCFA generation	[75,79]
Liposomes	Phospholipids from natural plant sources	Excellent biocompatibility; supports co-encapsulation of hydrophilic anthocyanins and lipophilic ingredients	Improves permeability and absorption of polar metabolites across intestinal epithelial membranes	[75,79]
Nanoemulsions	Composite systems of lipids and surfactants	Large interfacial surface area; high dispersion and clear optical properties in liquid systems	Overcomes solubility limits of poorly soluble phenolic polymers and enhances metabolic conversion at intestinal targets	[18,75]

## Data Availability

The data that support the findings of this study are available from the corresponding author upon reasonable request.

## Abbreviations

MBDD	Behavioral and developmental disorders
Nrf2	Nuclear factor erythroid 2-related factor 2
SOD	Superoxide dismutase
GPx	Glutathione peroxidase
ADHD	Attention-deficit/hyperactivity disorder
Trk	Tropomyosin receptor kinase
NDDs	Neurodevelopmental disorders
ASD	Autism spectrum disorder
ROS	Reactive oxygen species
TNF-α	Tumor necrosis factor-alpha
IL-1β	Interleukin-1 beta
